# Supplementary figures and images for: Microglial STAT1-sufficiency is required for resistance to toxoplasmic encephalitis
Source: PLoS Pathog. 2022 Sep 6;18(9):e1010637. doi: 10.1371/journal.ppat.1010637 (PMC9481170; doi:10.1371/journal.ppat.1010637)

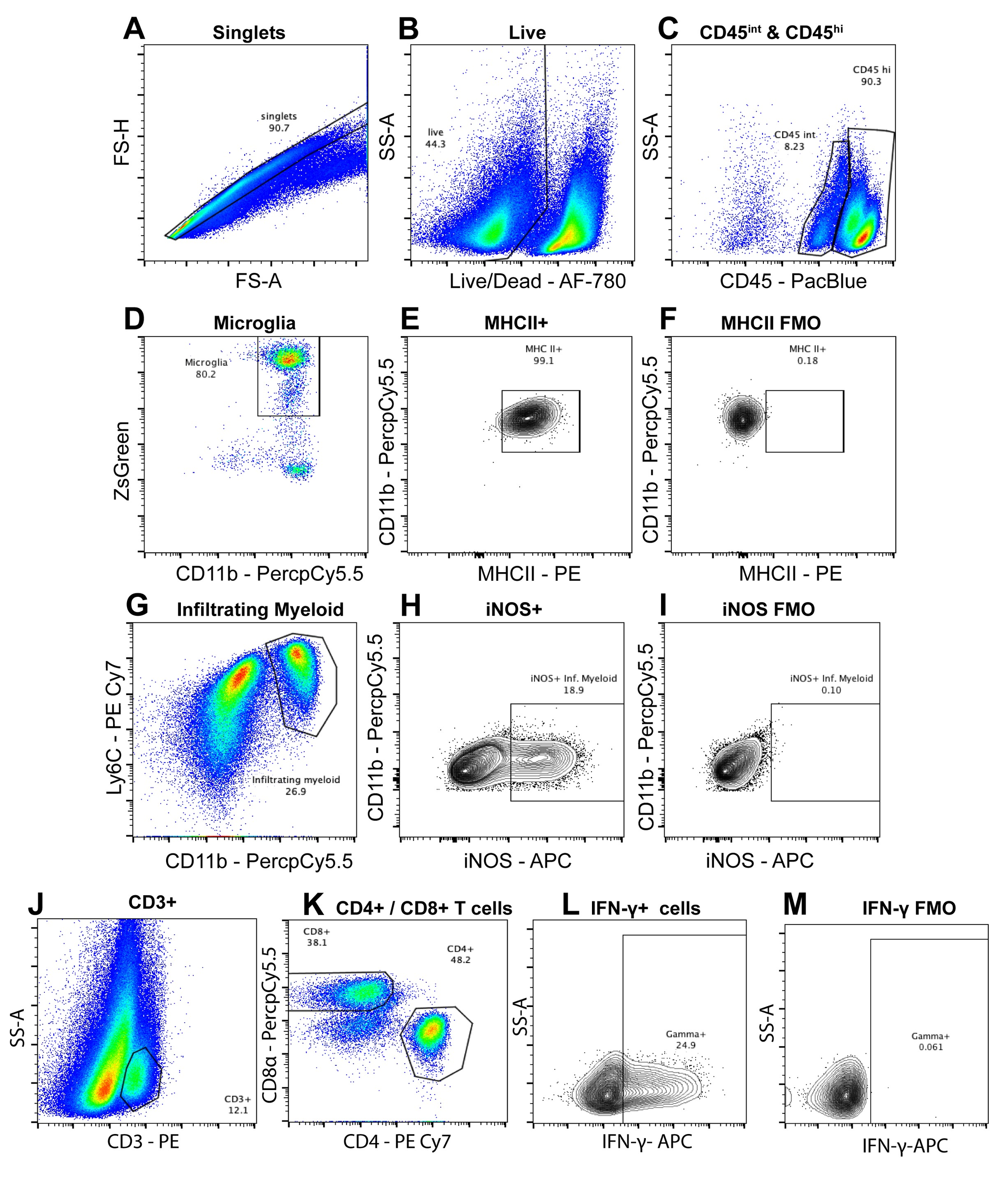

Supplement: S1 Fig — Myeloid and T cells isolated from mice with ZsGreen+ microglia were analyzed via flow cytometry. (A-C) For all panels, cells were pre-gated on singlets (A), then live cells using a viability dye (B). (C) Cells were gated to identify CD45 hi (brain-infiltrating) and CD45 int (brain-resident) immune cells. (D) Microglia were gated based on CD45 intermediate expression, ZsGreen and CD11b expression, and (E-F) MHC II positivity was assessed using FMO. (G) Infiltrating myeloid cells were gated based on CD45 hi expression, and the expression of both CD11b and Ly6C. (H-I) iNOS expression was determined via FMO gating. (J-K) T cells were gated based on the expression of CD3 and CD4 or CD8. (L-M) IFN-γ expression on CD3+CD4+ and CD3+CD8+ cells was determined based on FMO positivity. (TIF) [file ppat.1010637.s001.tif]

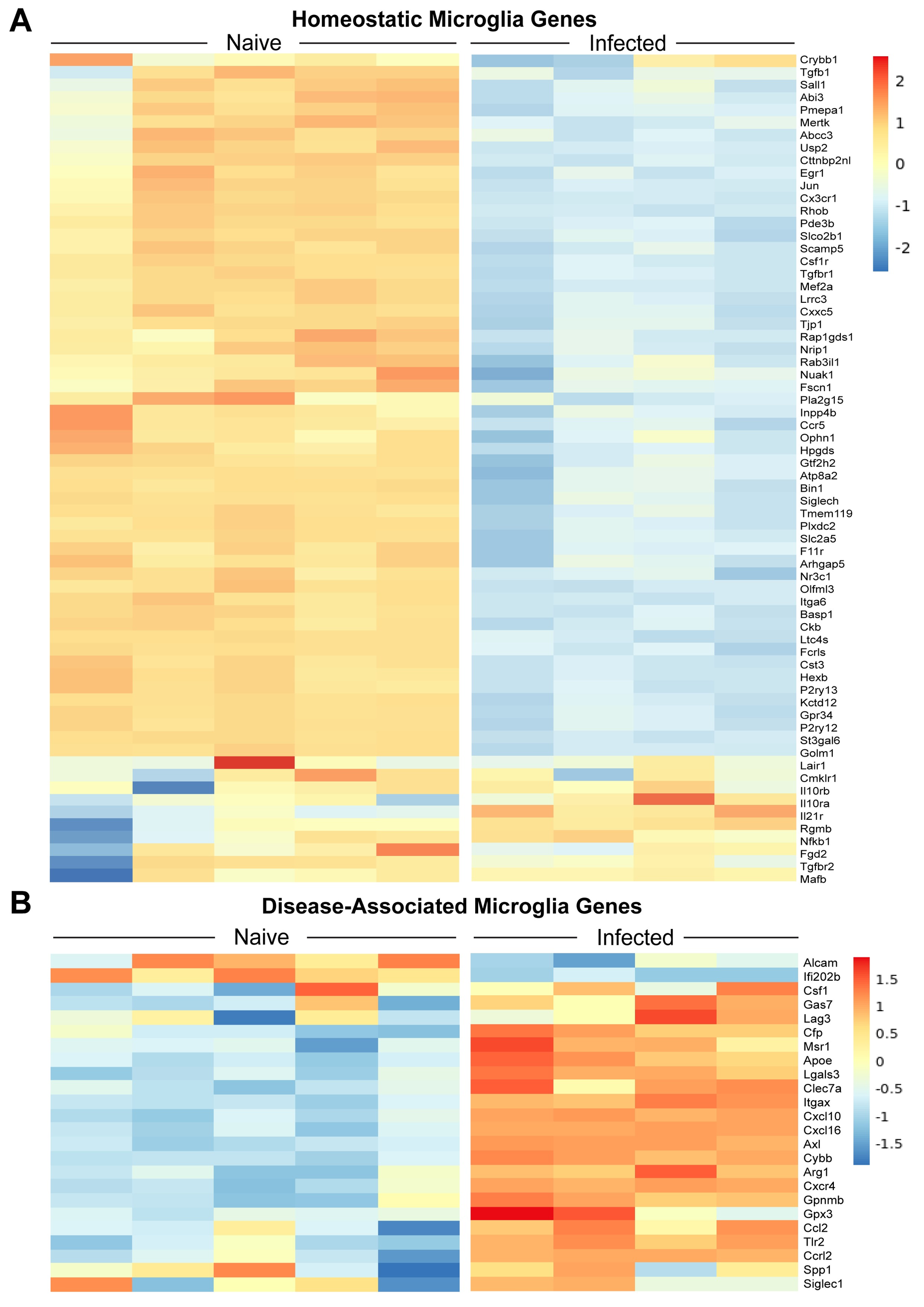

Supplement: S2 Fig — Microglia from wild-type naïve or wild-type mice infected with T. gondii for 4 weeks were FACS-sorted and RNA-sequenced at 4 weeks post-infection. (A-B) Heat maps displaying hierarchically-clustered gene expression from regularized log transformed gene abundance counts. Heatmap data display the full set of significantly differentially expressed microglial homeostatic genes (A), or disease-associated microglia genes (B), shared across neurodegenerative models investigated in Krasemann et al., 2017 and reflected in the naïve vs. T. gondii-infected DESeq2 dataset. Statistical significance was defined in the differential gene expression analysis as a BH adjusted p value < 0.05. n = 4-5 mice per group. (TIF) [file ppat.1010637.s002.tif]

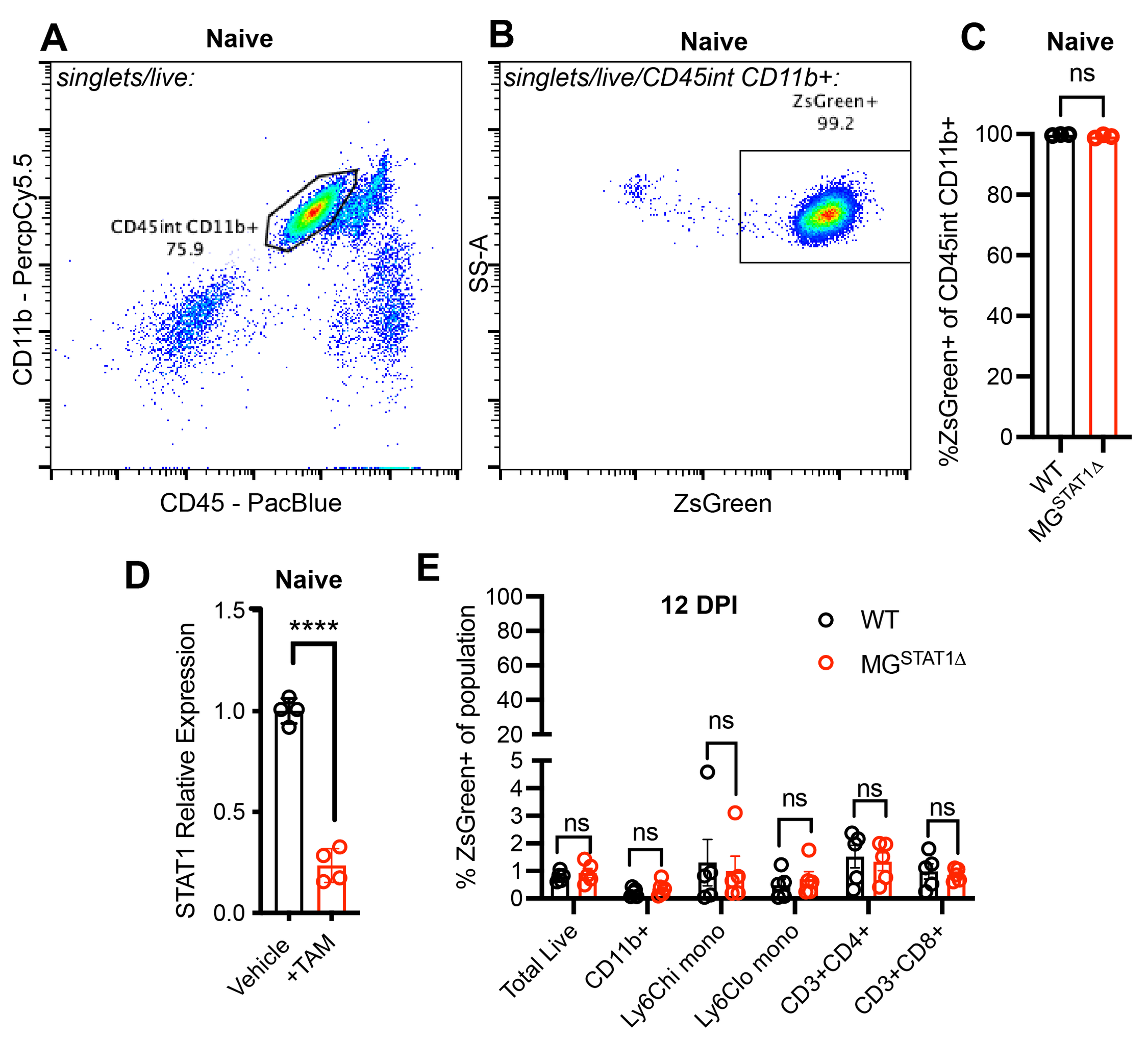

Supplement: S3 Fig — Naïve microglia and microglia isolated from brains at 12 DPI were analyzed by flow cytometry or RT-qPCR for relative gene expression. (A-B) Representative FACS plots indicating gating strategy for validating microglial ZsGreen expression in naïve mice. (C) Flow cytometric quantification of ZsGreen expression in total CD45int CD11b+ cells in naïve WT or MGSTAT1Δ mice. (D) RT-qPCR quantification of Stat1 relative expression in microglia that were magnetically enriched from naïve vehicle or tamoxifen (TAM)-treated MGSTAT1Δ mice. (E) Flow cytometric quantification of ZsGreen expression in various immune populations isolated from blood at 12 DPI, in WT or MGSTAT1Δ mice. Statistical significance was determined via unpaired t test, with n = 3-5 mice per group (C-E). ns = not significant; **** = p < 10-4. (TIF) [file ppat.1010637.s003.tif]

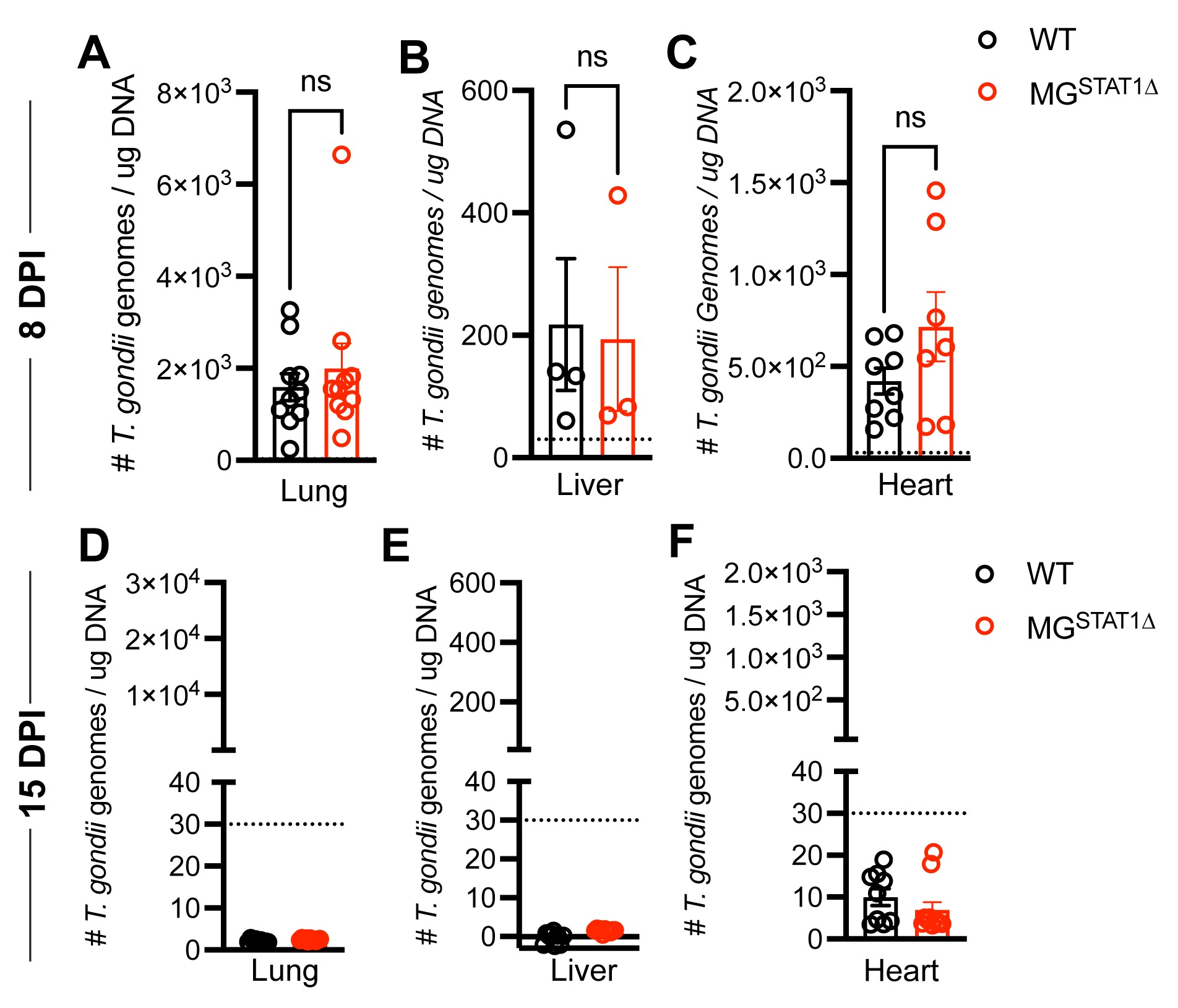

Supplement: S4 Fig — WT and MGSTAT1Δ mice were intraperitoneally infected with 10 cysts of the Me49 strain of T. gondii, and peripheral tissues were harvested and analyzed by qPCR for parasite genomic DNA, relative to total tissue DNA. Parasite burden was quantified at 8 DPI in lung (A), liver (B), and heart (C) tissue. Parasite burden was quantified at 15 DPI in lung (D), liver (E), and heart (F) tissue. (A-C) Statistical significance was determined via randomized block ANOVA using compiled data from 2-3 experiments with n = 10 mice per group (A), n = 7-8 mice per group (C), n = 9-11 mice per group (D-F), or via unpaired t test with n = 3-4 mice per group (B). Dotted line on y axis denotes assay limit of quantification, based on lower limit of standard curve. ns = not significant. (TIF) [file ppat.1010637.s004.tif]

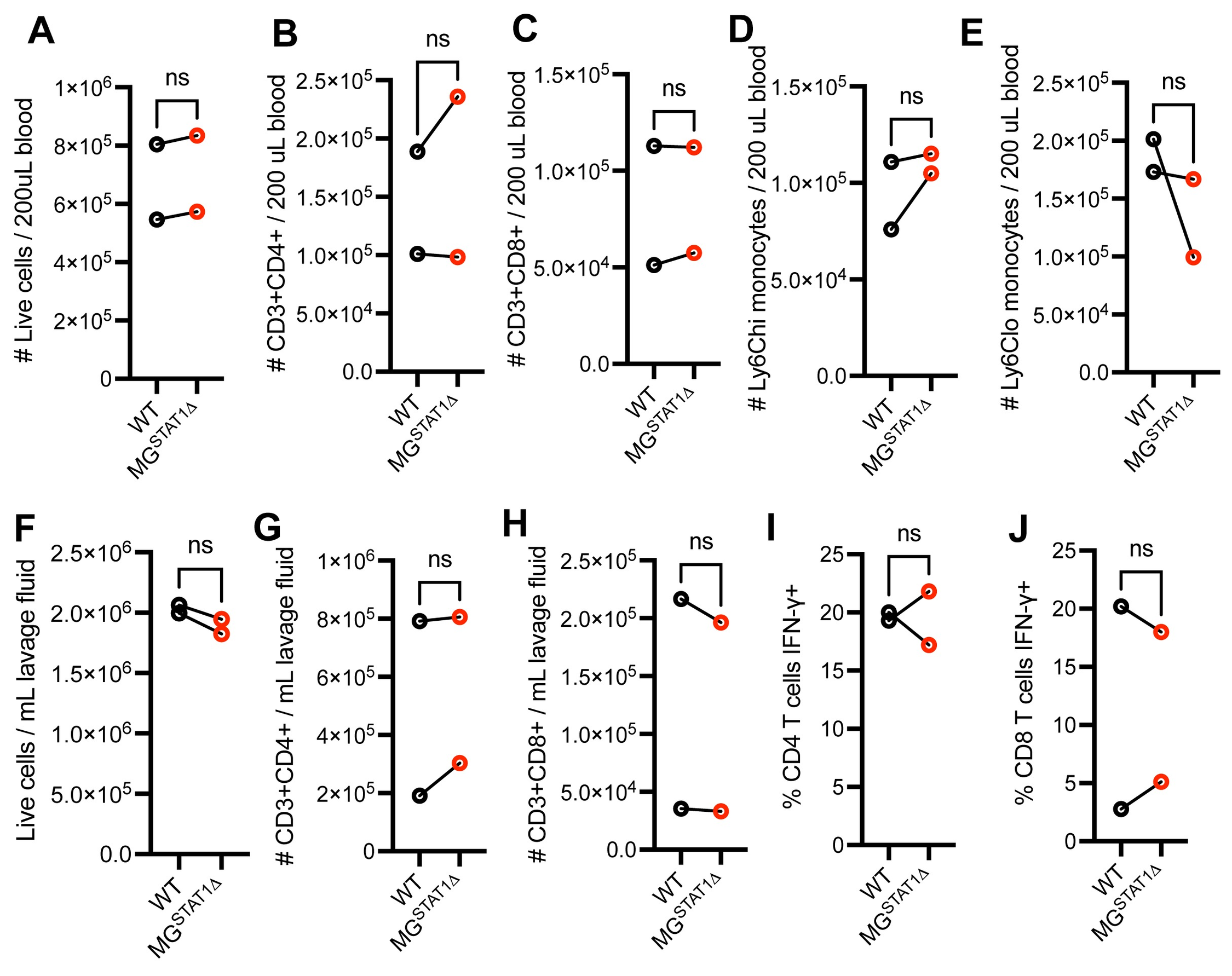

Supplement: S5 Fig — WT and MGSTAT1Δ mice were intraperitoneally infected with 10 cysts of the Me49 strain of T. gondii, and immune cells from blood and peritoneal fluid were analyzed by flow cytometry. (A-E) Flow cytometric quantification of total live immune cells (A), CD3+CD4+ T cell count (B), CD3+CD8+ T cell count (C), CD11b+Ly6Chi monocytes (D), and CD11b+Ly6Clo monocytes (E), calculated from blood. (F-J) Quantification of total live cells (F), number of CD3+CD4+ T cells, (G) number of CD3+CD8+ T cells (H), and CD4+ or CD8+ T cell expression of IFN-γ (I-J) isolated from the peritoneal cavity at 8 DPI. Statistical significance was determined by two-way randomized block ANOVA (A-J). ns = not significant, n = 11 per group from two pooled experiments (A-E), or n = 7-9 mice per group from two pooled experiments (F-J). Biological replicates are individual mice, with group means from individual experiments plotted as open circles with black lines connecting experimental and control groups. Source data (A-H) are provided in a source data file. (TIF) [file ppat.1010637.s005.tif]

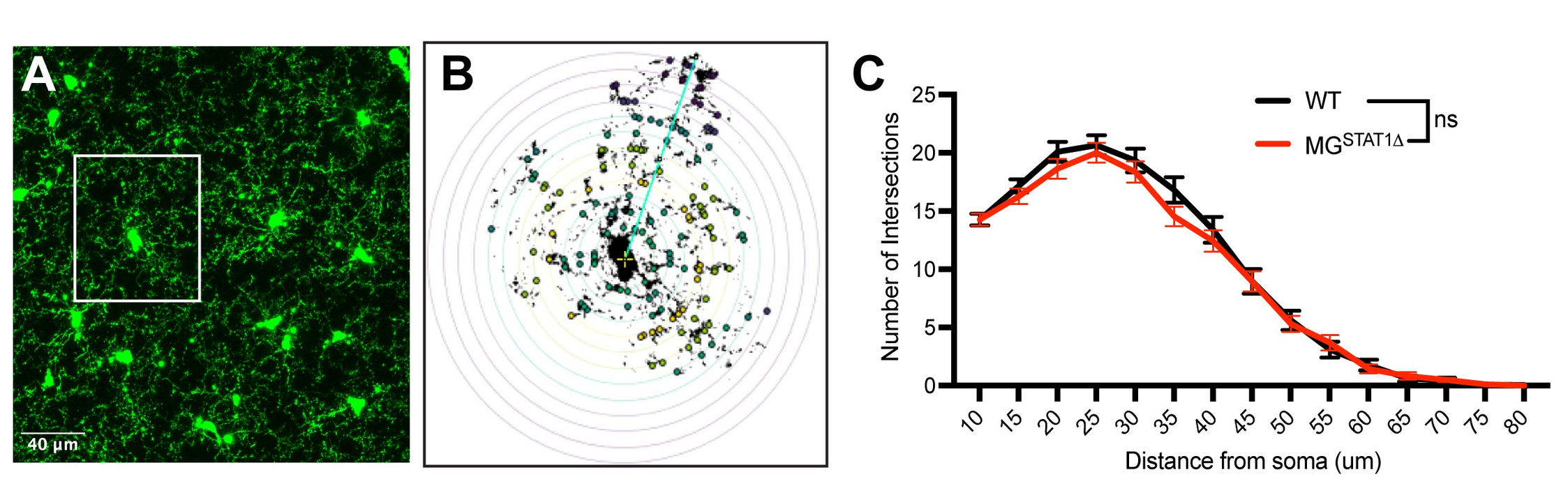

Supplement: S6 Fig — To analyze microglial activation, Sholl analysis was performed on microglia from the somato-motor cortex of naïve WT and MGSTAT1Δ mice. (A) ZsGreen+ microglia were imaged using a confocal microscope, and images were processed into a maximum projection using Fiji. (B) Images were made binary, microglia were manually isolated to determine cell process continuity, and the Sholl analysis Fiji plugin was executed to record intersections at varying soma distances. (C) Quantification of Sholl data via two-way ANOVA with Sidak’s multiple comparisons test, n = 64-67 microglia from 3 mice per group. (A) Scale bar = 40 μm. (TIF) [file ppat.1010637.s006.tif]

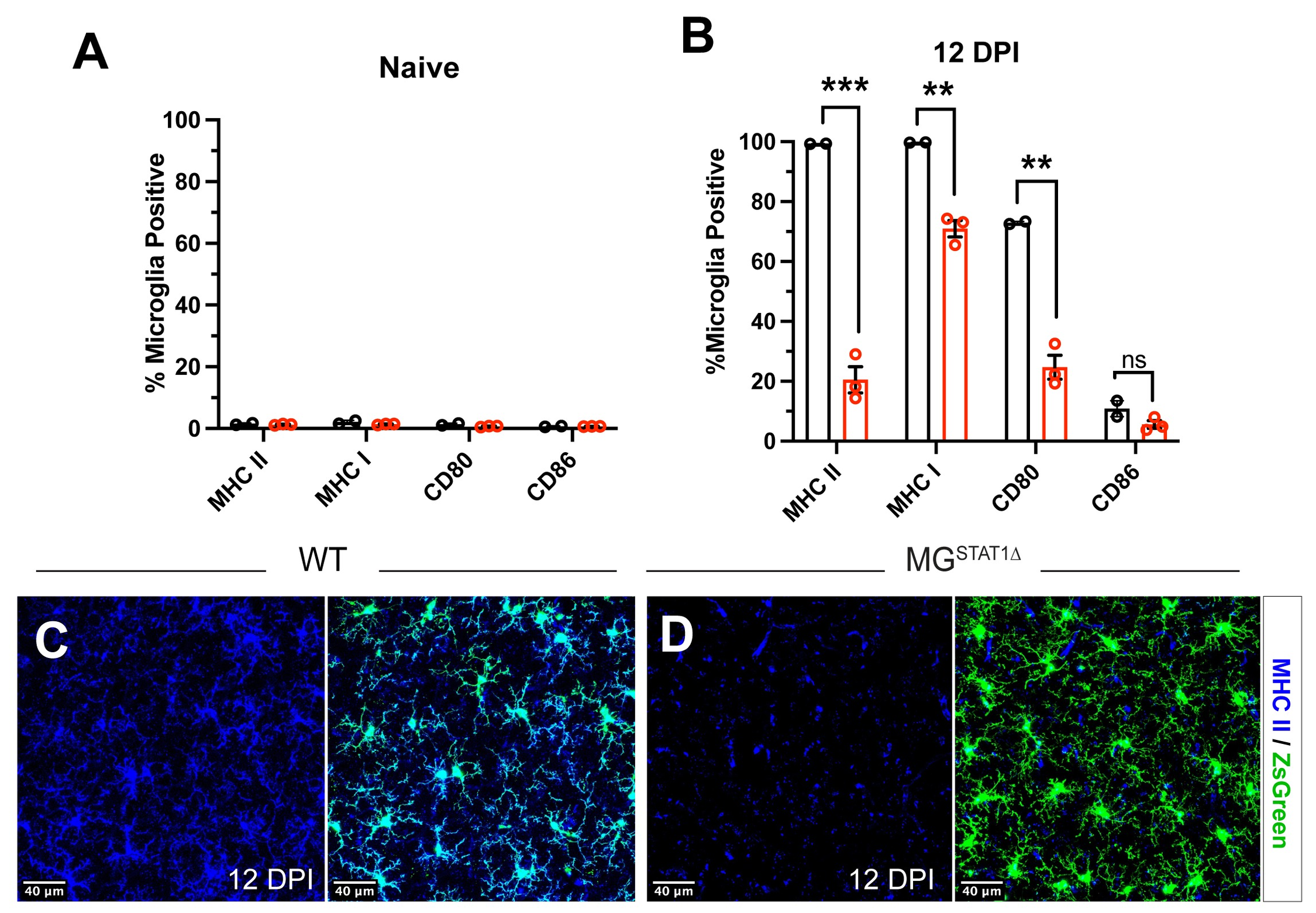

Supplement: S7 Fig — Microglia were isolated from naïve or 12 DPI infected WT or MGSTAT1Δ mouse brains and analyzed by flow cytometry and confocal microscopy. Flow cytometric analysis of microglial major histocompatibility complex and co-stimulatory molecules in (A) naïve brains, and (B) 12 DPI brains. (C-D) Immunohistochemical analysis of MHC II positivity by confocal microscopy; scale bar = 40 um, blue indicates MHC II, and green indicates ZsGreen fluorescence. n = 2-3 per group, unpaired t test (A-B). ns = not significant, ** = p <0.01, *** = p < 0.001. (TIF) [file ppat.1010637.s007.tif]

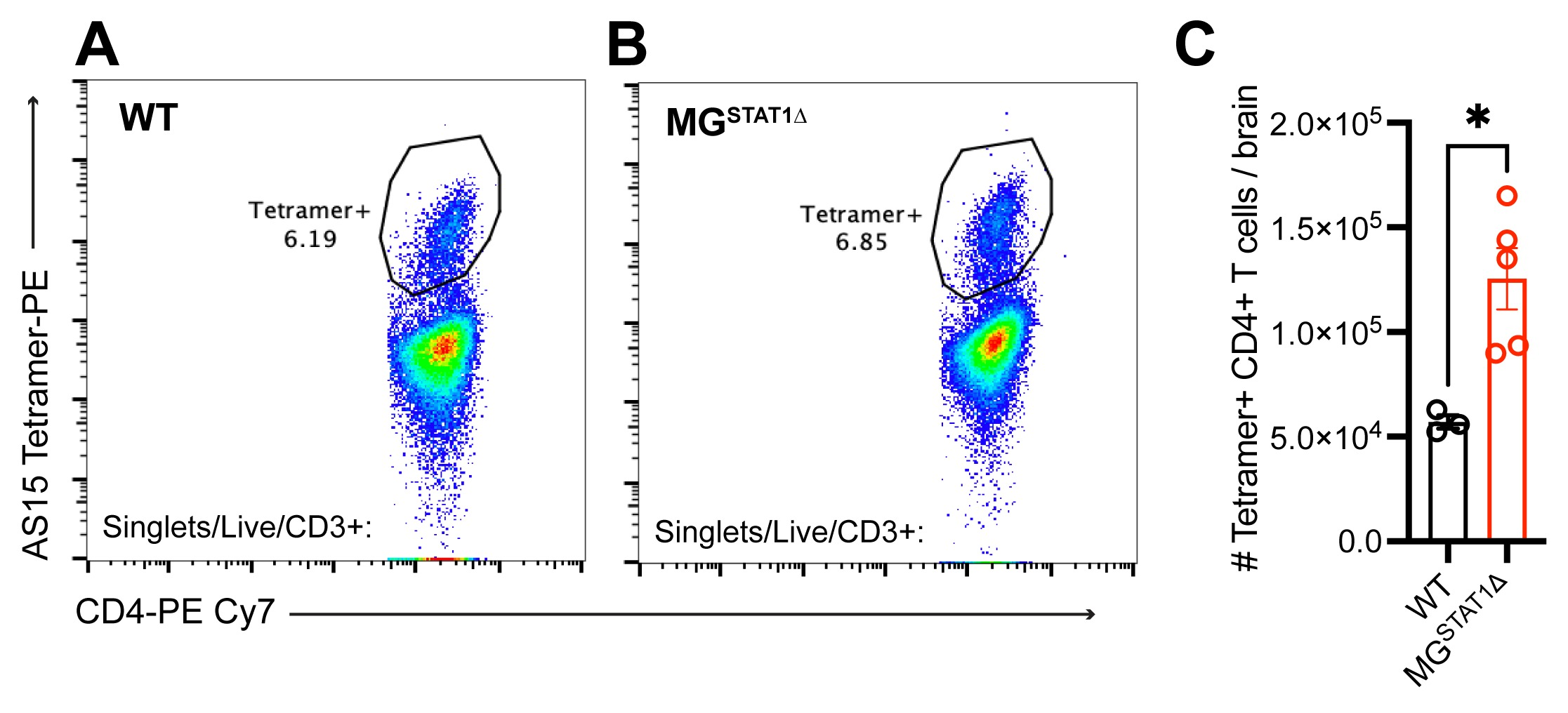

Supplement: S8 Fig — WT and MGSTAT1Δ mice were intraperitoneally infected with 10 cysts of the Me49 strain of T. gondii, and brain-infiltrating CD4+ T cells were analyzed by flow cytometry for MHC II I-Ab AS15 tetramer positivity at 15 DPI. (A-B) Representative FACS plots indicating tetramer gating in WT (A) and MGSTAT1Δ mice (B). (C) Quantification of CD3+CD4+ tetramer+ cells isolated from brains at 15 DPI via unpaired t test, n = 3 and 5 mice per group. * = p <0.05. Error bars indicate standard error of the mean. (TIF) [file ppat.1010637.s008.tif]

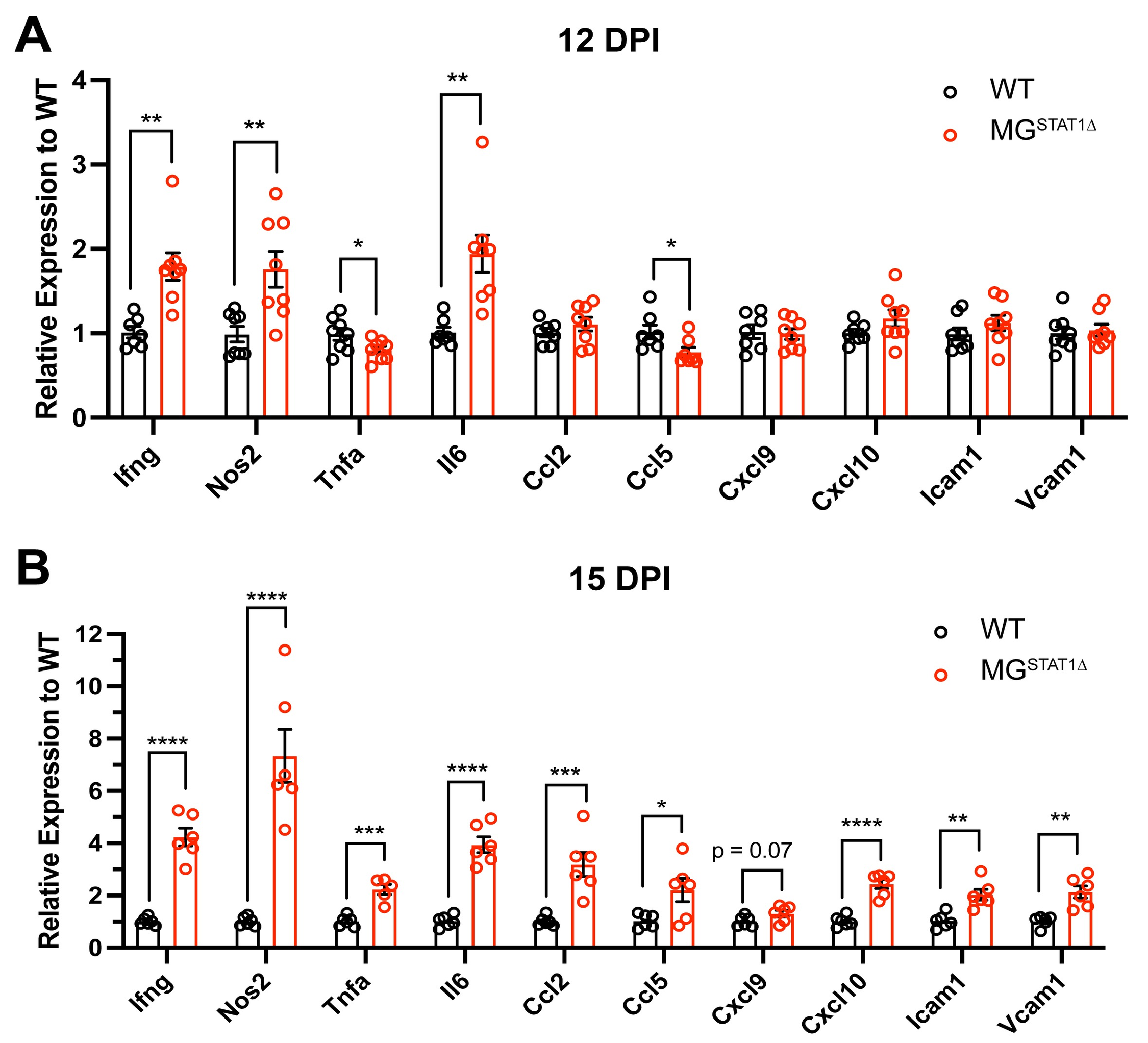

Supplement: S9 Fig — Whole brain homogenate from WT and MGSTAT1Δ mice was analyzed by RT-qPCR for a panel of various anti-parasitic genes. (A) 12 DPI and (B) 15 DPI immune effector profile. Statistical significance was determined by unpaired t test for one experiment (A), and two-way randomized block ANOVA from two pooled experiments (B). n = 8 per group (A), and n = 9-10 mice per group (B). * = p < 0.05, ** = p < 0.01, *** = p < 0.001, **** = p < 10-4. Error bars indicate standard error of the mean. (TIF) [file ppat.1010637.s009.tif]
