# Supplementary material for: Microglial STAT1-sufficiency is required for resistance to toxoplasmic encephalitis
Source: PLoS Pathog. 2022 Sep 6;18(9):e1010637. doi: 10.1371/journal.ppat.1010637 (PMC9481170; doi:10.1371/journal.ppat.1010637)
Supplement: S1 Table — (PDF) [file ppat.1010637.s010.pdf]

S1 Table. List of qPCR primers and probes used for analyzing *T. gondii* genomic DNA and RNA gene expression.

**Reagent**	**Sequence**
Toxo 529bp RE Forward Primer	5’-CAG TCC TGA TAT CTC TCC TCC AAG A-3’
Toxo 529bp RE Reverse Primer	5’- CAC AGA AGG GAC AGA AGT CGA A-3’
Toxo 529bp RE Probe	5’-/56-FAM/CTA CAG +A+CG+A+TGC C/3IABkFQ/-3’
Toxo Sag1 Forward Primer	5'-GGA TCG CCT GAG AAG CAT-3'
Toxo Sag1 Primer Reverse Primer	5'-ATG GAA ACG TGA CTG GCT-3'
Toxo Sag1 Probe	5'-/56-FAM/CT GTA CCG T/Zen/G CAA CTG GAG TTT GC/3IABkFQ/-3'
Toxo Bag1 Forward Primer	5'-CGT GGA GTT CGA CAG CAA-3'
Toxo Bag1 Reverse Primer	5'-ATA ACG ATG GCT CCG TTG TC-3'
Toxo Bag1 Probe	5'-/56-FAM/TT TGG CTG A/Zen/C TTG CCA GGT CTT CA/3IABkFQ/-3'
Toxo Act1 Forward Primer	5'-CGT GAG AGA ATG ACC CAG ATT AT-3'
Toxo Act1 Reverse Primer	5'-ACC GGA GGA GTA CAG AGA AA-3'
Toxo Act1 Probe	5'-/56-FAM/TC GAA ACC T/Zen/T TAA CGT CCC TGC CA/3IABkFQ/-3'
