# Supplementary material for: Microglial STAT1-sufficiency is required for resistance to toxoplasmic encephalitis
Source: PLoS Pathog. 2022 Sep 6;18(9):e1010637. doi: 10.1371/journal.ppat.1010637 (PMC9481170; doi:10.1371/journal.ppat.1010637)
Supplement: Attachment — Submitted filename: Reviewer rebuttal 7-30.pdf [file ppat.1010637.s011.pdf]

Part I - Summary
Please use this section to discuss strengths/weaknesses of study, novelty/significance, general execution and scholarship.

Reviewer #1 commented that "this article is well-written and the conclusions are well-supported by the data. I have only minor critiques for the authors to clarify before it is acceptable for publication." Reviewer #2 also shared, "This manuscript was a pleasure to review. It is well written, boasts extensive and convincing controls throughout, and is circumspect in the interpretation of its findings." The authors would like to thank both reviewers for their positive and constructive feedback. We greatly appreciate their recommendations for strengthening several areas of the manuscript. We are excited that our work has been overall positively received, and have made the following revisions to improve the quality of this manuscript for re-submission:

Part II – Major Issues: Key Experiments Required for Acceptance
Please use this section for editorial suggestions as well as relatively minor modifications of existing data that would enhance clarity.

(No major issues were identified.)

Part III – Minor Issues: Editorial and Data Presentation Modifications
Please use this section for editorial suggestions as well as relatively minor modifications of existing data that would enhance clarity.

Note: All line numbers refer to the "clean" manuscript, not the "Track Changes" version.

Reviewer #1 Comments:

1. Materials and Methods, Line 475: Is it correct that brains were embedded in formalin?

   We would like to thank Reviewer #1 for bringing this error to our attention. Brains were embedded in paraffin, not formalin, for H&E analysis (now Line 516).

2. Materials and Methods, Line 489: Authors refer to the concentration of Actinomycin D in digestion buffer as 45 μm and 3 μm. I believe this should probably be μM.

   This line has been corrected, with micromolar (μM) concentration as the appropriate unit of measurement (now Line 530).

3. Materials and Methods, Line 550: Authors refer to Supplemental Table 1, which I am not seeing included in the Supplemental File.

   We thank Reviewer #1 for bringing this to our attention. We included this table at the end of the main text body. Per revision guidelines, this table is now uploaded as a PDF under "Supporting Information."

4. Figure Legend 1: Please include number of mice per group that were analyzed. In other figure legends, the authors list the "total n". The format in which the data is presented it

is impossible to tell how balanced these groups are, and a number per group (or even a range) would be more informative (ie, n=6-7 per group rather than n=13).

We thank Reviewer #1 for raising the limitations in how the biological replicates, *n*, are formatted throughout the initial submission. We agree that breaking down total *n* by group is more informative, and we have revised each figure legend accordingly.

5. Figure 2: What is the time point post-infection for panel 2B?

   The time point for Figure 2B, 12 days post-infection, is now included in the figure legend.

6. Please note that the legend for Fig 6 is duplicated in the Supplemental Information.

   We apologize for this duplication, which is now resolved.

7. Supplemental Figure 3: is it correct that the flow cytometry plots shown are from a naïve mouse? I am surprised by how many CD45hi cells are present.

   Reviewer #1 is correct that the flow cytometry plots in S3A and S3B are shown from a naïve mouse, and we believe that the high number of CD45hi cells are likely a result of blood contamination. We note that via immunohistochemistry, we see few, if any immune cells present in the naïve brain. We thus speculate that this potential artifact may be due a combination of factors specific to our flow cytometry preps. The combined use of intermediate CD45 expression with our fluorescent reporter to gate microglia has allowed us to control for this technical limitation throughout our experiments.

8. Supplemental Figure 4: How is it that points are being measured below the limit of detection?

   We thank Reviewer #1 for noting this error. We have corrected the S4 Fig legend to state "limit of quantification," not "limit of detection." This limit indicates the lowest valid measurement of our standard curve in the qPCR assay (30 genomes), and thus reflects the lowest limit by which CT values can be quantified with reasonable statistical accuracy.

9. Authors interchange "Supplemental" and "Supplementary" in both the main text and the Legends.

   We apologize for this inconsistency. All figure/table names, captions, and in-text citations have been reformatted in accordance with PLOS Pathogens guidelines (i.e. "S1 Fig", rather than "Supplemental/Supplementary Figure 1").

<u>Reviewer #2 Comments:</u>

1. The authors seem to argue that microglial STAT1 deletion is primarily influencing disease by preventing the activities of IFN-g signaling, though of course the actions of other interferons would also be impacted. The authors should discuss this possibility and its potential relevance to their findings, if any.

We thank Reviewer #2 for raising this point, and agree that it warrants discussion. We hypothesize that amongst additional cytokine drivers of STAT1 signaling, defective IFNAR-STAT1 signaling may potentially play a compounding role in our study. The literature on STAT1 activation by other cytokines such as Il-27 and Il-6 is fairly mixed and their effects vary based on cell type and cross-talk mechanisms (Heinrich et al., 2003; Haan et al., 2005; Kalliolias et al., 2008; Baker et al., 2010). We address these themes in Lines 382-398, focusing on IFNAR signaling:

> *While STAT1 is activated downstream of multiple interferons and IFN-γR activation was not directly investigated in the current study, multiple lines of evidence support the hypothesis that a defect in the ability for microglia to respond to IFN-γ is the primary driver of the pathology observed in MG$^{STAT1\Delta}$ mice during T. gondii challenge. IFN-γ is essential during both acute and chronic T. gondii infection, with STAT1-/- mice closely phenocopying IFN-γ-/- mice in uniformly succumbing during the acute phase of infection* [1]*, and pharmacological depletion of IFN-γ specifically during chronic infection similarly resulting in uniform lethality* [2]. *In contrast to IFN-γ, IFN-α/β appear to carry a more limited protective role during T. gondii infection* [1,3]. *In an oral model of infection, global genetic deletion of their shared receptor, IFNAR, results in increased brain parasite burden but more modest levels of animal mortality (~50% mortality in IFNAR-/- vs. ~20% mortality in wild-type controls by 50 DPI)* [3]. *Together, this literature supports a predominant role for IFN-γ-STAT1 signaling in the current study. However, future experiments will be necessary to determine to what extent IFN-α/β, or other potential activators of STAT1 such as Il-27 and Il-6* [4–7]*, could play compounding roles or are independently capable of shaping the microglial response to T. gondii infection. Lastly, our RNA-sequencing data did not suggest appreciable levels of IFN-λR on microglia, suggesting limited relevance of type III interferons to this cell type [data publicly available through GEO accession code GSE203655].*

2. The finding that T cell responses are "potentiated" in microglial-stat1 knockouts seems likely to be an indirect effect of increased parasite burden, rather than a specific consequence of microglial stat1 deletion. This should be discussed (unless I've missed it).

   The authors agree with Reviewer #2 that increased T cell responses in MG$^{STAT1\Delta}$ mice are likely not due to an effect that microglia are exerting directly on T cells, but due to indirect effects of increased parasite burden in the brain. This topic is now discussed in Lines 434-447, which includes a discussion of a new tetramer experiment (comment #3):

   > *While the precise determinants of tachyzoite relative to bradyzoite enrichment in MG$^{STAT1\Delta}$ mice remain unclear, the observation that brain-infiltrating T cell and myeloid responses are increased in MG$^{STAT1\Delta}$ relative to control mice may best be explained as a consequence of increased brain parasite burden, rather than a direct result of microglial STAT1 deletion. Increased parasite burden due to an inability of microglia to restrict lytic parasite replication would be expected to lead to increased T cell stimulation due to an increase in antigen availability in the brain that can be presented by infiltrating myeloid cells. Consistent with this view, we observed an increase in T. gondii antigen-specific CD4+ T cells in the brain, measured by flow cytometry using an MHC II tetramer, as well as increased CD4+ and CD8+ T cell IFN-γ production.*

Moreover, increased parasite replication and egress, a lytic process [reviewed in ref. 8], may also increase release of inflammatory signals such as alarmins that drive further recruitment of immune cells from circulation [9,10]. *The inability of a robustly activated brain-infiltrating immune compartment to compensate for microglial STAT1 deficiency thus underscores the necessity for STAT1 signaling in brain-resident populations during T. gondii challenge.*

3. Related to above - were antigen-specific T cell responses assessed? Can the authors use a tetramer staining approach to more robustly assess the T gondii-specific T cell response, to distinguish it from an influx of non-specific inflammatory cells. For the measurements of IFN-g in T cells, were cells restimulated ex-vivo using a T. gondii peptide? Some clarification of this issue is warranted. Data would be welcome, but not necessarily required.

We thank Reviewer #2 for raising this point related to antigen-specific T cell responses. We have added data using a tetramer specific for the *T. gondii* peptide AS15 as an additional supplementary figure (now S8 Fig). Previous literature has identified AS15 peptide as contributing to brain parasite control (Grover et al., *Infection & Immun*ity, 2012). Details on the staining method are now included in Lines 548-550.

These new data indicate no significant differences in CD4 tetramer frequency between groups, but reveal a statistically significant increase in CD4 tetramer number that tracks with the total increased number of CD4 cells present in the brains of $MG^{STAT1\Delta}$ mice at 15 DPI. These data indicate that antigen-specific T cells are present in large number in the brains of $MG^{STAT1\Delta}$ mice. A description of these data are included in Lines 262-265, and they have been integrated into the Discussion (#2, above) in Lines 434-447.

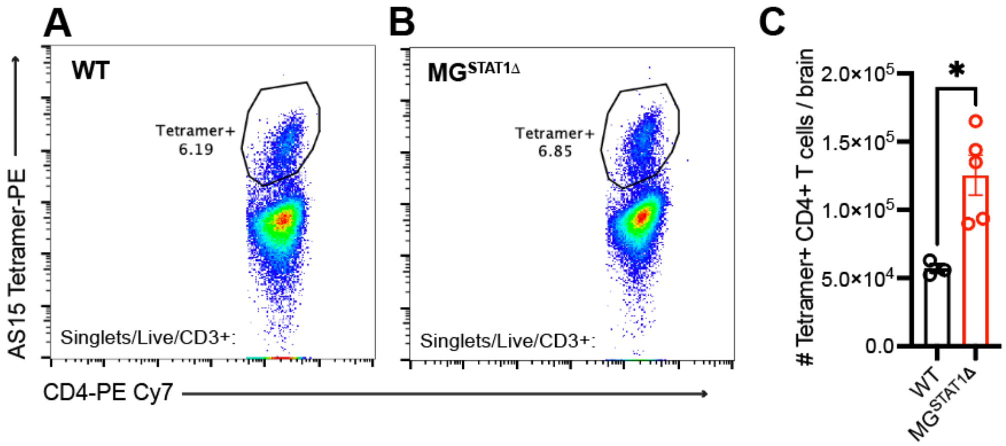

For the measurements of IFN-γ in T cells isolated from both brain and peritoneal lavage, cells did not receive any additional stimulation *ex-vivo*. In Fig 4G-4H and S5I – S5J Fig, cells were solely treated with the golgi membrane inhibitor Brefeldin A in order to collect data that most closely reflects endogenous IFN-γ expression. This point is now emphasized in the Methods section (Lines 554-555).

References:

***Highlighted references appear as new additions to the manuscript text. Non-highlighted references were included in initial submission.*

1. Lieberman LA, Banica M, Reiner SL, Hunter CA. STAT1 Plays a Critical Role in the Regulation of Antimicrobial Effector Mechanisms, but Not in the Development of Th1-Type Responses during Toxoplasmosis. J Immunol. 2004;172: 457–463. doi:10.4049/jimmunol.172.1.457
2. Gazzinelli R, Xu Y, Hieny S, Cheever A, Sher A. Simultaneous depletion of CD4+ and CD8+ T lymphocytes is required to reactivate chronic infection with Toxoplasma gondii. J Immunol. 1992;149: 175–180.
3. Matta SK, Olias P, Huang Z, Wang Q, Park E, Yokoyama WM, et al. Toxoplasma gondii effector TgIST blocks type I interferon signaling to promote infection. Proc Natl Acad Sci. 2019;116: 17480–17491. doi:10.1073/pnas.1904637116
4. Heinrich PC, Behrmann I, Haan S, Hermanns HM, Muller-Newen G, Schaper F. Principles of interleukin (IL)-6-type cytokine signalling and its regulation. Biochem J. 2003;374: 1–20. doi:10.1042/bj20030407
5. Haan S, Keller JF, Behrmann I, Heinrich PC, Haan C. Multiple reasons for an inefficient STAT1 response upon IL-6-type cytokine stimulation. Cell Signal. 2005;17: 1542–1550. doi:10.1016/j.cellsig.2005.03.010
6. Kalliolias GD, Ivashkiv LB. IL-27 Activates Human Monocytes via STAT1 and Suppresses IL-10 Production but the Inflammatory Functions of IL-27 Are Abrogated by TLRs and p38. J Immunol. 2008;180: 6325–6333. doi:10.4049/jimmunol.180.9.6325
7. Baker BJ, Park KW, Qin H, Ma X, Benveniste EN. IL-27 inhibits OSM-mediated TNF-α and iNOS gene expression in microglia. Glia. 2010;58: 1082–1093. doi:10.1002/glia.20989
8. Blader IJ, Coleman BI, Chen C-T, Gubbels M-J. Lytic Cycle of Toxoplasma gondii : 15 Years Later. Annu Rev Microbiol. 2015;69: 463–485. doi:10.1146/annurev-micro-091014-104100
9. Batista SJ, Still KM, Johanson D, Thompson JA, O'Brien CA, Lukens JR, et al. Gasdermin-D-dependent IL-1α release from microglia promotes protective immunity during chronic Toxoplasma gondii infection. Nat Commun. 2020;11: 3687. doi:10.1038/s41467-020-17491-z
10. Still KM, Batista SJ, O'Brien CA, Oyesola OO, Früh SP, Webb LM, et al. Astrocytes promote a protective immune response to brain Toxoplasma gondii infection via IL-33-ST2 signaling. Blader IJ, editor. PLOS Pathog. 2020;16: e1009027. doi:10.1371/journal.ppat.1009027

Formatting / Editorial changes:

We have made several minor changes to the manuscript to format it according to PLOS Pathogens guidelines:

- An Author Summary has been added between the Abstract and Introduction.
- In-text citations for all figures have been reformatted (i.e. "Fig 1B-1F" instead of "Fig. 1B-F", and "S1A-S1D Fig" instead of "Supplementary Fig. 1A-D).
- Figures have been removed from the end of the main body text and have been submitted as separate files for re-submission, per guidelines. Figure legends remain at the end of the main manuscript document.

- Supplemental Information: S8 Fig was formerly a qPCR analysis of whole brain RNA. With the addition of the CD4 tetramer response, this now appears as S9 Fig.
- Supplemental Information: S1 Table legend appears at the end of the end of the document. The accompanying table has been uploaded separately as a PDF.
- Funding information has been removed from the Acknowledgments sections, per guidelines.
- Transparent boxes were added around each figure to allow a small amount of white space surrounding images when they were converted from .ai to .tiff files (in the event cropping is performed during the editorial process). Slight adjustments to spacing between panels were performed to center figures in Adobe Illustrator.
